# Old hematopoietic stem cells retain competence to reconstitute a youthful B cell system that is highly responsive to protein-based vaccination

**DOI:** 10.1186/s12979-025-00507-x

**Published:** 2025-04-05

**Authors:** Paul Kunath, Dominik Pflumm, Bettina Moehrle, Vadim Sakk, Alina Seidel, Jan Münch, Hartmut Geiger, Reinhold Schirmbeck

**Affiliations:** 1 Department of Internal Medicine I, University Hospital of Ulm, Ulm, Germany; 2 Institute of Molecular Medicine, Ulm University, Ulm, Germany; 3 Institute of Molecular Virology, Ulm University Medical Center, Ulm, Germany

**Keywords:** Hematopoietic stem cells, Old immune system, B cells, SARS-CoV-2 spike vaccine, IgG antibody response

## Abstract

**Background:**

Ageing-associated remodeling of the murine B cell system is accompanied with a reduction of CD19^+^ B cells such as follicular B cells (FOB) and an accumulation of age-associated B cells (ABC) or activated B cell subsets. This remodeling is thought to confer an attenuated antibody response, such as to SARS-CoV-2 spike (S) vaccines in both aged mice and humans. To gain insight into the *de novo* development and function of an old B cell system, we reconstituted young and old immune systems by transferring hematopoietic stem cells (HSCs) from immune-competent young (2–3 months) CD45.1^+^ donors (DY-HSC) or old (20–24 months) donors (DO-HSC) into T and B cell-deficient young recipient CD45.2^+^ RAG1^−/−^ mice, followed by protein-based vaccination.

**Results:**

In the same environment of young RAG1^−/−^ mice, transplanted DO-HSCs compared to DY-HSCs reconstituted lower numbers of CD19^+^ B cells and CD45.1^+^ cells, though the engraftment of donor-derived HSCs in the young bone marrow (BM) was very similar. Furthermore, indicative for youthful and unchallenged B cell systems, and in contrast to aged mice, very low levels of antigen-experienced memory B cells or age-associated B cells (ABC) developed in both DY-HSC and DO-HSC hosts. The commercially available recombinant SARS-CoV-2 S vaccine (NVX-CoV2373) induced lower IgG^+^ S-antibody titers and pseudovirus neutralization activity in old compared to young mice. In contrast, very similar high IgG^+^ S-antibody titers were induced in DO-HSC and DY-HSC hosts, and pseudovirus neutralization activity was even enhanced in DO-HSC compared with DY-HSC hosts.

**Conclusions:**

Both DO-HSCs and DY-HSCs established in the young recipient BM to a similar extend, suggesting that the concomitant reduction in the *de novo* reconstitution of CD19^+^ B cells in DO-HSC vs. DY-HSC transplanted animals is specifically related to old HSCs. DO-HSCs and DY-HSCs reconstitute very similar unchallenged B cell systems that efficiently elicit antigen-specific IgG antibodies by protein-based vaccination. Old HSCs thus retain competence to reconstitute a youthful and functional B cell system, at least in the young environment of transplanted RAG1^−/−^ mice. This suggests that it is primarily age-related factors, and not HSCs per se, that influence the composition and functionality of the old B cell system.

**Supplementary Information:**

The online version contains supplementary material available at 10.1186/s12979-025-00507-x.

## Introduction

Reduced B and T cell responses, particularly to newly encountered antigens, are common in older adults as compared to younger individuals [1, 2]. As part of the ageing-associated remodeling of the immune system, molecular deficiencies in older B cells have been reported to impair foreign antigen presentation and thus directly lower T cell-dependent antibody responses [3–7]. Data from the SARS-CoV-2 pandemic, but also from seasonal influenza virus infections, showed that older adults have a reduced responsiveness to vaccination associated with lower antibody titers and a faster decline of the titers [1, 8–17]. Strategies to improve vaccine efficacy in old people are currently limited to approaches like increasing the antigen concentration or the vaccination frequency [11, 12, 14, 18–21]. However, also the antigen structure (e.g., trimeric versus monomeric antigens) and/or different heterologous prime/booster regimens (e.g., DNA/prime and protein/booster) might be sufficient to unleash a full function of the old immune system to elicit a more young-like antibody response [22–27].

Ageing-associated remodeling of the immune system is driven by aging of hematopoietic stem cells (HSCs) and the bone marrow (BM) niche, which, in combination with thymic involution and changes in secondary lymphoid structures [28], result in a marked reduction in lymphopoiesis in the old [29, 30]. There was also an intrinsic accumulation of immunoregulatory cells such as CD4^+^ Tregs [31], memory-phenotype CD8^+^ T cells [32] and/or age-associated B cells (ABC) [2, 4], which likely contribute to the reduced number and especially to the reduced function of B cells in the old. To study the quality of naturally aged versus *de novo* reconstituted young and old immune systems, we have established a HSC-transplantation model in T and B cell-deficient young RAG1^−/−^ hosts [28]. In this model, T cell systems in RAG1^−/−^ mice transplanted with old HSCs (from 20 to 24 months old animals) induced a significantly reduced antigen-specific CD8^+^ T cell response upon vaccination in comparison to RAG1^−/−^ mice transplanted with young HSCs (from 2 to 3 month old animals) [28]. Overall, these and other studies implied that aged HSCs determine individual HSC-derived aged T cell subsets in an antigen independent manner [28, 33, 34].

To determine the extent of the aging-associated remodeling of the B cell system on the response to vaccination in more detail, we employ here again the HSC-transplantation model in T and B cell-deficient young RAG1^−/−^ hosts. We investigate whether and to what extent young and old B cell systems, reconstituted from young and old HSCs, induce serum antibody responses after vaccination. We quantify SARS-CoV-2 spike (S)-specific antibody titers and their neutralization capacity using an established SARS-CoV-2 pseudovirus infection platform [27, 35]. The major advantage of this model is to compare the HSC-driven reconstitution of B cells under standardized conditions, as young and old donor HSCs are exposed to the same young microenvironment, and that all B cells (and also T cells) exclusively reconstitute from transplanted HSCs [28]. The experimental setup allows us therefore to identify HSC-imprinted but also HSC-independent B cell subsets in comparison to analyzing directly aged mice.

## Materials & methods

### Mice

Young (2–3 months) and old (22–24 months) female CD45.1/STEM [36] and C57Bl/6 mice were obtained from our in-house breeding colonies (SFB 1506 ´Ageing at interfaces´; project Z02) at the Tierforschungszentrum of Ulm University. All experiments were performed in accordance to the National Animal Welfare Law and approved by the Committee on the Ethics of Animal Experiments of the University of Ulm and the Regierungspräsidium Tübingen, Germany. Mice were routinely housed in our animal facility under specific pathogen free (SPF) conditions. Housing conditions include a temperature range of 22 +/- 1 °C, a relative humidity of 55 +/- 10%, an air change rate of 15 times and a light/dark change of 12/12 hrs. For nutrition a ssniff M-Z autoclavable complete feed for mice-breeding (# V1124-3) and water ad libitum were supplied. Aging mice were routinely checked for overall appearance, weight-loss and appearance of injuries. We for the first time established a stringent monitoring of CD8 T cell subsets in the blood to identify healthy ageing of mice: The reciprocal decline in naive (T_N_) and increase in memory CD8 T cells, like antigen-naïve virtual (T_VM_) and antigen-experienced true memory (T_TM_) CD8 T-cell frequencies in ageing mice is a hallmark of ageing [37]. Most prominent upon ageing, the proportion of naïve (CD44^−^, CD49d^int^) CD8 T_N_ in the peripheral blood declined from about 70–80% in young (2–3 months) to about 5–20% in old (20–24 months) mice, whereas antigen-experienced true memory CD8 T_TM_ increased from 3 to 10% up to 15–30% CD8 T_TM_. Mice with a strikingly enhanced frequency (≥50%) of antigen-experienced CD8 T_TM_ in peripheral blood, indicative for aging-associated diseases and cancer, were excluded from analyses.

### Transplantation of HSCs into RAG1^−/−^ mice

Female young (2–3 months) B6.129S7-Rag1tm1Mom/J (RAG1^−/−^) mice were used as recipients in the HSC-transplantation experiments [28]. Briefly, HSCs were isolated from the respective CD45.1^+^ young (DY-HSC) and old donors (DO-HSC) and sorted as Lin^−^ Sca-1^+^ c-kit^+^ CD34^−^ Flk-2^−^ cells from the bone marrow (BM) using a BD FACS Aria III (BD Bioscience) (Fig.S1). Six hundred HSCs were transplanted into sublethally (6.5 Gy) irradiated mice (GSR D1 self-shielding gamma irradiator using the nuclide Cs^137^ with an activity radiation source of 87 TBq). Alternatively, mice were pretreated with Busulfan (15 mg/kg; Busulfex, #697507, Otsuka America Pharmaceutical; USA) in 200 µl PBS for five consecutive days, followed by HSC transplantation at day seven. Reconstitution of donor-derived immune cells was monitored in the peripheral blood at 6 to 18 weeks and finally in the spleen at 18 weeks post transplantation. To quantitatively analyze immune cells in aged mice and the HSC-transplantation model, we sacrified mice at indicated times to determine cell numbers and frequencies in the spleen. Therefore, we set up different experiments for analyzing B cells in non-immunized mice and for vaccine-induced antibody responses (see below).

### Immunization of mice

HSC recipients (at 18 weeks post transplantation) and ageing mice were immunized subcutaneously at day 0 (prime) and day 22 (boost) with 1 µg of the recombinant SARS-CoV-2 S vaccine Nuvaxovid (NVX-CoV2373, Novavax, USA) that contained Matrix-M adjuvants. Blood samples were collected on day 14 after the boost injection.

### Determination of S-specific antibody titers

S-specific IgG^+^ antibody titers were determined by a quantitative ELISA. Briefly, Nunc MaxiSorp 96-well plates (Thermo Fisher Scientific, USA) were coated with 0.1 µg/well of a recombinant S6-P_ΔTM/EPEA_ detection antigen [27]. Plates were washed (0.05% Tween-20 in PBS) and experimental serum samples from immunized and non-immunized control mice were added (1:4.000 or 1:12.500) in blocking buffer (3% BSA in PBS) for 30 min at room temperature. Plates were washed followed by incubation with a secondary HRP-conjugated goat anti-mouse Ig (1:2.000; # 550337, BD Biosciences, USA) and developed with 0.4 mg/ml OPD (#P6787, Sigma-Aldrich, USA) in substrate solution buffer (0.05 M citrate-phosphate buffer, 0.012% hydrogen peroxide, pH 5.0). Reaction was stopped with 5% of sulfuric acid. Plates were analyzed with a plate reader Spectra MAX (MWG Biotech, Germany) at 492 nm. For quantification of S-specific IgG titers, a commercially available primary antibody directed against the SARS-CoV-2 S2 protein domain (#944302, Biolegend, USA) served as calibration standard. The calibration curve was calculated using a “sigmoidal, 4PL, X is concentration” interpolation model in GraphPad Prism (GraphPad, USA). The absolute S-specific IgG titers (ng/µl) were calculated based on the equation of the calibration curve (x = c_*_(((a-d)/(y-d))-1)^^(1/b)^, a = bottom, b = hillslope, c = IC_50_, d = top, y = OD_492_ value). Furthermore, S-specific IgG^+^ antibody titers were determined by standard endpoint ELISA as described previously [27]. The antigen-specific endpoint titers were defined as the highest serum dilution that resulted in an absorbance value three times greater than that of control sera from unimmunized young or old mice.

### Pseudovirus neutralization assay

Use of the antibody-mediated SARS-CoV-2 spike-specific pseudovirus neutralization assay was described previously [27].

### Flow cytometry (FCM)

For characterization of different types of B cells in the peripheral blood and spleen, we performed immunostainings according to standard protocols, using anti-CD19/APC (#115512; BioLegend), anti-CD21/35/Pacific Blue (#123414, BioLegend), anti-CD23/PE-Cy7 (#101614; BioLegend), anti-CD38/PE-efluor610 (#61-0381-82, Invitrogen), anti-CD45R(B220)/FITC (#103206, BioLegend), anti-CD95/Biotin (13-0951-85, Invitrogen) x Streptavidin/Brilliant Violet 605 (#405229, BioLegend), anti-CD138/PE (#142504, BioLegend), anti-IgD/SuperBright 702 (#67-5993-82, Invitrogen) and anti-IgM/APC-Cy7 (#406516, Biolegend). For a Dump channel, we used anti-CD3/Alexa Fluor 700 (#152316, BioLegend), anti-F4/80/Alexa Fluor 700 (#1231130, Biolegend) and anti-GR1/Alexa Fluor 700 (#108422, BioLeged) antibodies. Samples were analyzed by flow cytometry (FCM) using an Attune NxT flow cytometer equipped with a four laser configuration (i.e., violet 405 nm, blue 488 nm, yellow 561 nm, red 637 nm) with fourteen colors and sixteen parameters (Thermo Fisher Scientific) and FlowLogic version 8.7 software (Inivai Technologies, Melbourne, Australia).

### Statistical analysis

The GraphPad Prism 9.4.1 software (GraphPad, San Diego, CA, USA) was used for statistical analyses and creation of graphs. Statistically significant differences between two indicated groups were usually determined using student’s unpaired *t*-test. P-values smaller than 0.05 were considered as statistically significant and indicated with asterisks in the graphs (*p* < 0.05*, *p* < 0.01** and *p* < 0.001***). Depicted data and group sizes are stated in the figure legends.

## Results

### Transplanted RAG1^−/−^ mice show HSC-dependent and HSC-independent reconstitution of B cell subsets

HSCs, from which all cells of the adaptive immune system originate, play a crucial role in the ageing-associated remodeling of the immune system that impairs its functional integrity, resulting in an increased susceptibility to infections and decreased responsiveness to vaccines in the elderly [1, 2, 38]. In this study, we want to answer if and how HSCs have an impact on the ageing-associated remodeling of the B cell system and its response to vaccination.

**Fig. 1:**
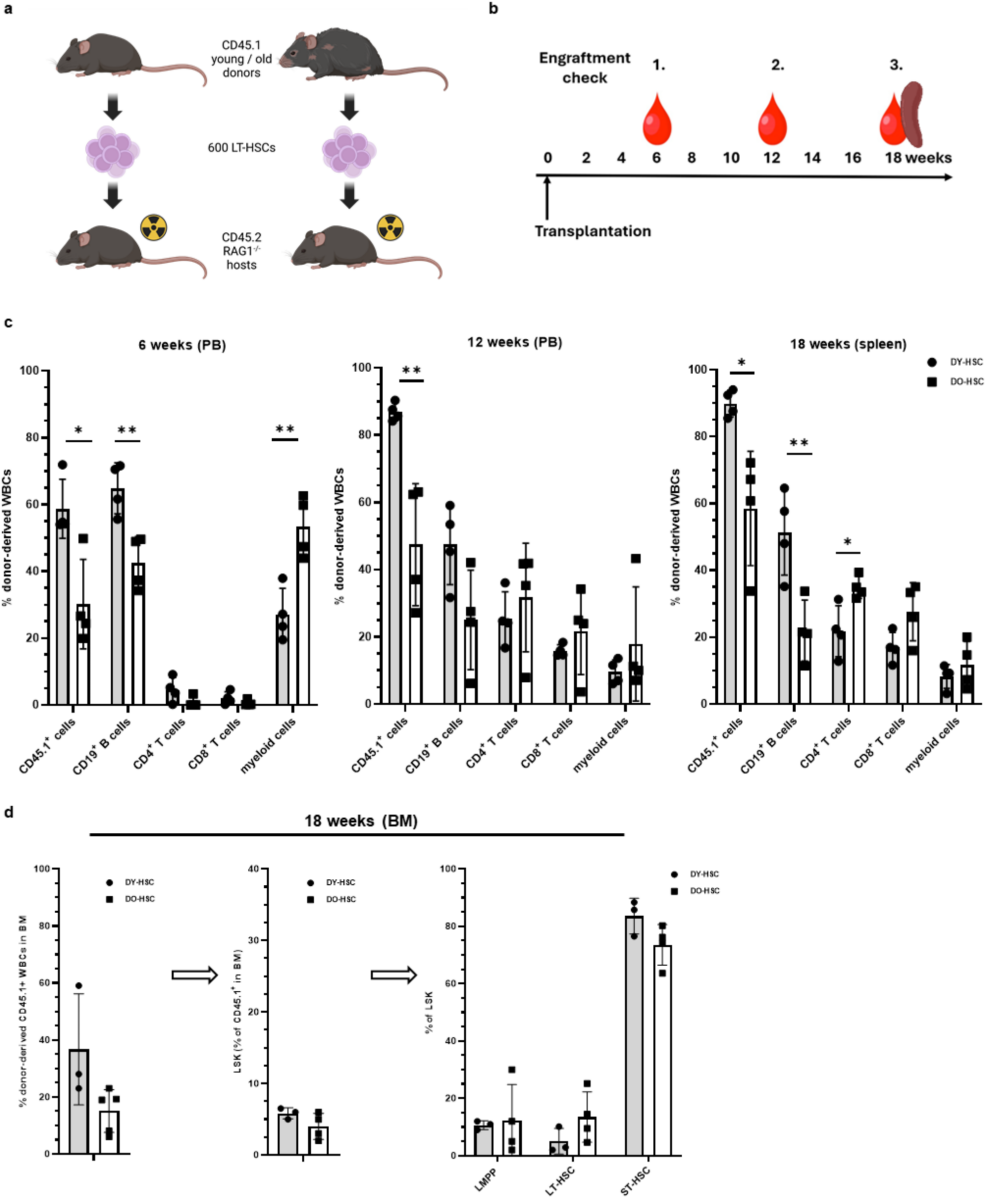
Engraftment of HSC-donor cells and immune cells in transplanted RAG1^−/−^ hosts. **(a)** An equal number of 600 LT-HSCs, isolated from young or old CD45.1/STEM donor mice, were transplanted into irradiated young CD45.2^+^ RAG1^−/−^ recipients. **(b)***De novo* reconstitution of immune cells usually was monitored at 6 and 12 weeks post transplantation in the peripheral blood (PB) and at 18 weeks post transplantation in the spleen. **(c)** HSCs from young (DY-HSC) (*n* = 4) or old (DO-HSC) mice (*n* = 4) were transplanted into irradiated young CD45.2^+^ RAG1^−/−^ recipients and the kinetics of cell reconstitution was analyzed. The percentage of donor-derived CD45.1^+^ cells, CD19^+^ B cells, CD4^+^ T cells, CD8^+^ T cells and CD45.1^+^ myeloid cells to total white blood cells (WBCs) was determined at 6, 12 and 18 weeks post transplantation by flow cytometry. **(d)** HSCs from young (DY-HSC) (*n* = 3) or old (DO-HSC) mice (*n* = 4) were transplanted into irradiated young CD45.2^+^ RAG1^−/−^ recipients and the frequencies of donor-derived CD45.1^+^ WBCs (left panel) and its Lin(-)/Sca-1(+)/c-Kit(+) LSK cell fraction (middle panel) was analyzed in the bone marrow (BM) at 18 weeks post transplantation by flow cytometry. Furthermore, the LSK cell pool was analyzed for lympho-myeloid primed progenitors (LMPPs), short-term HSCs (ST-HSCs) and long-term HSCs (LT-HSCs) (right panel). Statistical significance between the individual cell populations was determined using the unpaired students t-test. *p* < 0.05*, *p* < 0.01** If not indicated differences were not significant. Mean values±SD are shown. Created with Biorender.com

To analyze the *de novo* reconstitution of a young and old B cell systems under standardized conditions, we transplanted HSCs from immune-competent young and old CD45.1^+^ donor mice into T and B cell-deficient young CD45.2^+^ RAG1^-/-^ hosts (Fig. 1a) and analyzed the specific reconstitution of donor-derived cells [28]. Kinetic analyses (Fig. 1b) confirmed an old-specific skewing of HSC-derived cells toward the myeloid compartment in donor old HSCs (DO-HSC)- but not in donor young HSCs (DY-HSC)- recipients at early times (6 weeks) post transplantation (Fig. 1c). At this stage of transplantation, the reconstitution of CD45.1^+^ cells and CD19^+^ B cells from DO-HSCs was significantly reduced as compared to DY-HSCs, and T cell reconstitution was not yet efficiently developed (Fig. 1c). During the further course of reconstitution (12 to 18 weeks), the reduced reconstitution of CD45.1^+^ cells and CD19^+^ B cells from DO-HSCs vs. DY-HSCs was stable, but with the upcoming reconstitution of T cells the skewing of HSC-derived cells toward the myeloid compartment was no longer evident (Fig. 1c). Noticeable, very similar frequencies of T cells developed in hosts transplanted with DY-HSCs or DO-HSCs, suggesting that the young environment, like the reactivated young thymus in the RAG1^-/-^ recipients [28], facilitated the development of T cells from DO-HSCs.

At 18 weeks post transplantation, the overall reconstitution of CD45.1^+^ cells from DY-HSCs reached about 90% compared to about 60% of that from DO-HSCs (Fig. 1c). Alongside, the actual proportion of CD19^+^ B cells was reduced from about 50% in DY-HSC recipients to about 20% in DO-HSC recipients (Fig. 1c). Interestingly, we observed a similar pattern of DY-HSC vs. DO-HSC-mediated CD45.1^+^ and CD19^+^ B cell reconstitution using two very different methods to prepare host mice for HSC transplantation (Fig. S2). In particular, we compared busulfan treatment instead of standard irradiation prior to transplantation [39–41] (Fig S2), because it was widely unknown if irradiation and its possible side effects like acute early term inflammatory responses [42, 43] influence transplantation/reconstitution efficacy of LT-HSCs [44]. Our findings thus suggested that the level of inflammation induced by irradiation (if any) does not influence the quality of HSC-driven reconstitution of young and old immune systems and indicated a very stable HSC transplantation and immune cell reconstitution in young RAG1^-/-^ hosts that primarily depend on the age of HSCs.

To exclude that the differences in the CD19^+^ B cell reconstitution reflect a possible suboptimal establishment the DO-HSCs in the young recipient bone marrow (BM), we analyzed HSC engraftment in the BM. The frequency of CD45.1^+^ donor-derived cells was slightly decreased in the BM of DO-HSC vs. DY-HSC recipients (Fig. 1d). These DY-HSC and DO-HSC-derived CD45.1^+^ cells contained equal frequencies of Lin(-)/Sca-1(+)/c-Kit(+) LSK cells, and within these LSK cells also equal frequencies of lympho-myeloid primed progenitors (LMPPs), short-term HSCs (ST-HSCs) and long-term HSCs (LT-HSCs) (Fig. 1d). A trend towards even an elevated frequency of LT-HSCs was evident in DO-HSC recipients, as previously shown by us and others [45, 46]. These data thus excluded a suboptimal establishment of old HSCs in a young BM.

**Fig. 2:**
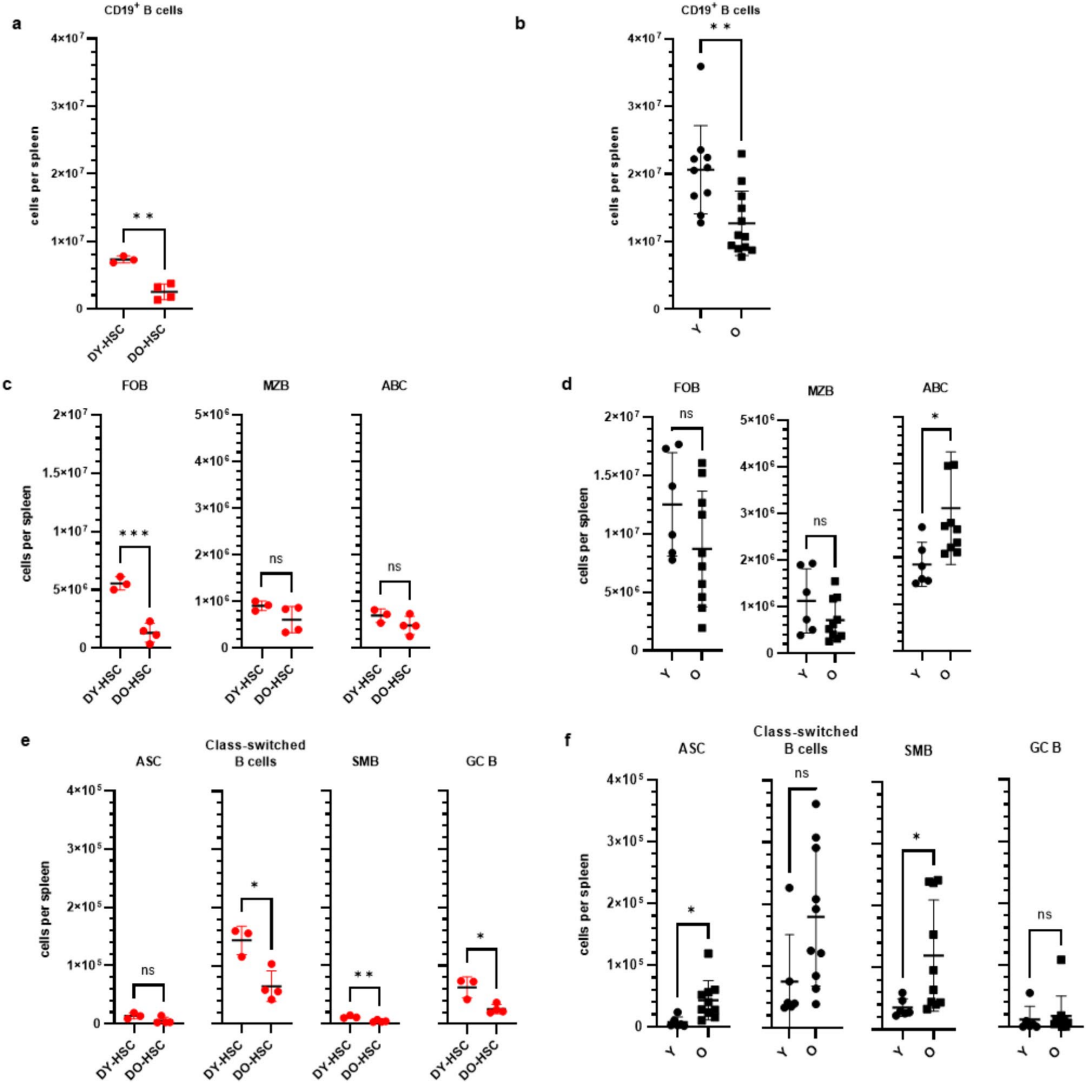
B cell subsets in naturally aged vs. HSC-transplanted mice. The number of CD19^+^ B cells in the spleen of DY-HSC and DO-HSC transplanted hosts at 18 weeks post transplantation (*n* = 6) **(a)** and of young (Y; 3 months) and old (O; 22–24 months) mice (*n* = 10–12) **(b)** was determined by flow cytometry. **(c-f)** The numbers of different B cell subsets such as follicular B cells (FOB), marginal zone B cells (MZB), age-associated B cells (ABC) **(c**,** d)**, and of IgD^−^ B cell subsets such as antibody secreting B cells (ASC), class switched B cells, germinal center B cells (GC B) and switched memory B cells (SMB) **(e**,** f)** was determined in the spleen of DY-HSC and DO-HSC hosts (*n* = 3–4) **(c**,** e)** and of young (Y) and old (O) mice (*n* = 6–10) **(d**,** f)** and was determined by flow cytometry. A representative HSC-transplantation experiment out of three experiments is shown in panels a, c and e. Statistical significance between the indicated B cell populations was determined using the unpaired students t-test. *p* < 0.05*, *p* < 0.01**, *p* < 0.001***. ns, not significant. Mean values±SD are shown

We next separately determined *de novo* HSC-mediated reconstitution of young and old B cell systems and closely associated B cell types (Fig. S3) in RAG1^−/−^ hosts as compared to the naturally B cell development in ageing mice. The number of splenic CD19^+^ B cells was reduced in DO-HSC compared to DY-HSC transplanted animals (Fig. 2a). Interestingly, also the naturally developed B cell system showed an overall decrease of the CD19^+^ B cell number in the spleen of old (≥20–24 months) compared to young (2–3 months) mice (Fig. 2b). In particular, the number of splenic CD19^+^ B cells in HSC-transplanted hosts reached about 10–15% in DO-HSC recipients and 35–40% in DY-HSC recipients at 18 weeks post transplantation as compared with the numbers in old and young mice, respectively (Fig. 2a, b). Similar to the B cell landscape in aged mice, the reduced reconstitution of splenic CD19^+^ B cells was accompanied by a decrease in the number of follicular B cells (FOB) that is more pronounced in DO-HSC vs. DY-HSC hosts (Fig. 2c, d). The number of marginal zone B cells (MZB) did not differ in DO-HSC and DY-HSC hosts as well as in young and old mice (Fig. 2c, d). In contrast, the number of age-associated B cells (ABC), representing a B cell subset that develops through continuous antigen exposure [4], significantly increased in old compared to young mice, but was very similar in DO-HSC compared to DY-HSC hosts (Fig. 2c, d). These findings were also confirmed adjusting the respective cell numbers to the different reconstitution efficacy of CD45.1^+^ WBCs in DO-HSC vs. DY-HSC hosts at 18 weeks post transplantation (Fig. 1c, Fig. S4). In conclusion, these findings show that young and old HSCs produce very low levels of ABCs, indicating that the accumulation of ABCs in old vs. young mice primarily proceeds in a HSC-independent manner [4].

As expected, the number of activated IgD^−^ B cells such as antibody-secreting plasma cells/plasma blasts (ASC), class-switched B cells, or switched memory B cells (SMB) was higher in old compared to young mice, whereas their numbers were either similar or slightly reduced in DO-HSC compared to DY-HSC hosts (Fig. 2e, f, Fig. S4). Consistently, lower numbers of these B cell subsets were found in DO-HSC hosts as compared to old mice (Fig. 2e, f). In contrast, higher numbers of germinal center (GC) B cells, established in DY-HSC and DO-HSC hosts as compared to ageing mice (Fig. 2e, f), suggesting that they might be spontaneously activated to some extent in the HSC-recipients by unknown mechanisms. These findings thus suggest that both young and old HSCs reconstitute very similar youthful and unchallenged B cell systems. In conclusion, the accumulation of activated IgD^−^ B cell subsets such as ASC or SMB in old mice proceeds in an HSC-independent manner.

To further analyze the status of the B-cell differentiation tree in naturally aged vs. HSC-transplanted mice, we determined the relative frequencies of the respective B cell subsets within the CD19^+^ B cell pool or, depending on the marker profile of ASCs (Fig. S3), within the activated B cell pool. In accordance with the analysis of absolute cell numbers (Fig. 2d, f), the relative frequencies of FOBs decreased, whereas the frequencies of ABCs, class-switched B cells, SMBs and ASCs increased in an age-dependent manner in old vs. young mice (Fig. S5). Furthermore, the frequency of FOBs significantly decreased in DO-HSC vs. DY-HSC recipients (Fig. S5). While the number of MZBs and ABCs was not affected in DO-HSC vs. DY-HSC recipients (Fig. 2c), particularly the frequency of MZBs was increased in DO-HSC recipients (Fig. S5). The frequency of GC cells was not affected by aging and not affected by the age of the transplanted HSCs, while their overall frequency slightly increased in HSC transplanted vs. naturally aged mice (Fig. S5). Similarly, the frequencies of SMBs, class-switched B cells and ASCs were not significantly differentially affected by the age of transplanted HSCs (Fig. S5). In particular, the increase in the frequencies of SMBs and ASCs in aged mice was not seen in DO-HSC vs. DY-HSC transplanted mice (Fig. S5). These analyses provide a more granular picture of ageing-associated changes in the *de novo* developed vs. naturally developed B-cell compartment, though we believe that the actual B cell numbers provide the most informative values about the actual composition of the B cell system in the HSC transplantation model, especially as there was equal reconstitution of aged and young progenitor/stem cells in the BM (Fig. 1d).

### Old HSCs retain competence to restore an immune-competent B cell compartment

**Fig. 3:**
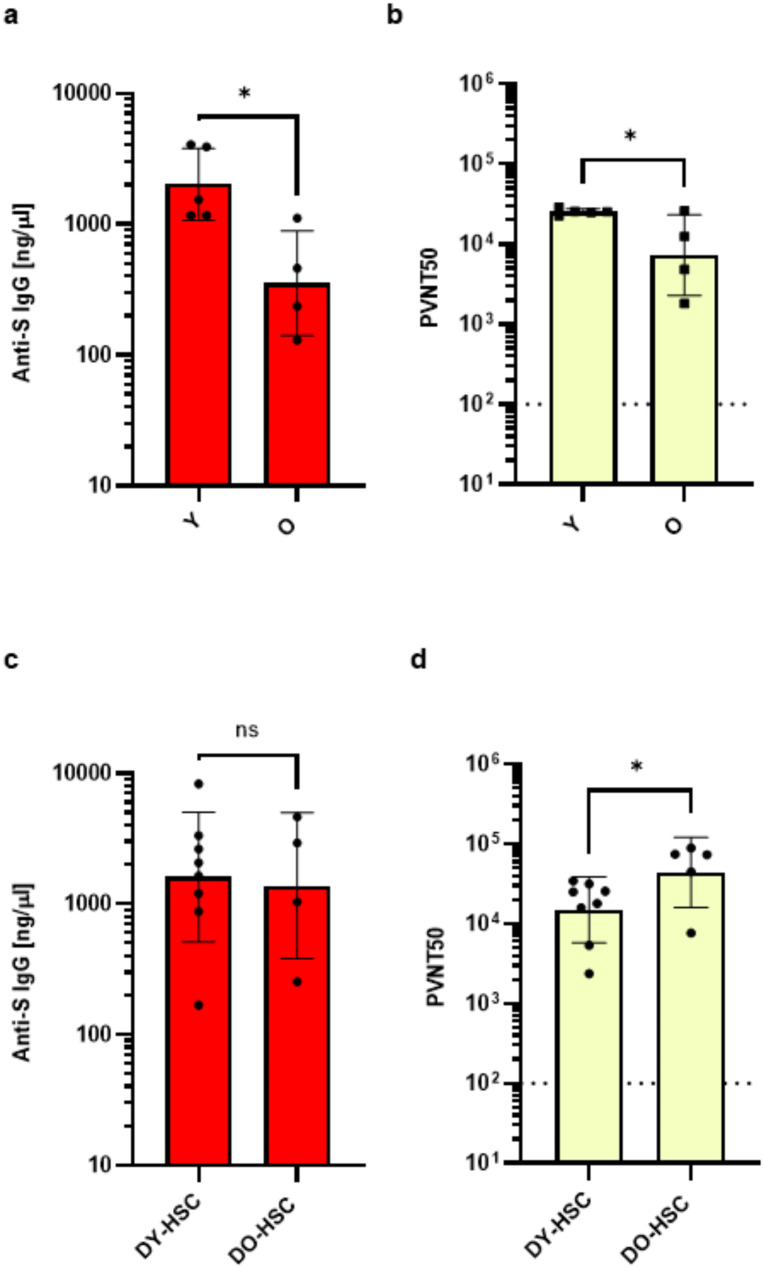
Priming of functional S-specific IgG antibodies in aged vs. HSC-transplanted hosts. Young (2–3 months) and old (22–24 months) mice (*n* = 4–5) **(a**,** b)**, and DY-HSC and DO-HSC-recipient mice at 18 weeks post transplantation (*n* = 5–8) **(c**,** d)** were immunized subcutaneously twice (at day 0 and 22) with 1 µg recombinant S-antigen (Novaxovid). **(a**,** c)** Serum samples were collected 14 days after the second immunization and tested in a quantitative S-specific IgG ELISA (anti-S IgG ng/µl) as described in M&M and **(b**,** d)** in a vesicular stomatitis virus (VSV)-based SARS-CoV-2 S-carrying pseudovirus model to determine the neutralizing activity of vaccine-induced antibodies. The results are depicted as serum dilution factors that result in 50% pseudovirus neutralization (PVNT50). Dotted lines represent the detection limit of PVNT50 values (100). Data are derived from two independent experiments and presented as geometric mean±SD. Statistical significance between the groups were determined using the unpaired student’s t-test (*p* < 0.05*). ns, not significant. Mean values±SD are shown

To determine the function of HSC-reconstituted B cell systems, we immunized DY-HSC and DO-HSC transplanted mice at 18 weeks post transplantation, and young and old immune-competent control mice with a recombinant Nuvaxovid (NVX-CoV2373) SARS-CoV-2 spike (S) vaccine [47–50], followed by a second injection after d22. Blood samples were collected two weeks after the boost injection. To determine anti S IgG antibodies, we established a novel quantitative ELISA that allows us to precisely determine antibody titers. Consistent with the decreased functional integrity of an old immune system [1, 2], vaccination induced significant lower IgG^+^ S-specific antibody titers in old compared to young mice (Fig. 3a). As expected, the higher level of IgG^+^ S-specific antibodies in young compared to old mice correlated with a significant better level of pseudovirus neutralization (Fig. 3b). In contrast, vaccination induced very similar titers of IgG^+^ S-specific antibodies in DO-HSC and DY-HSC hosts (Fig. 3c). This central finding was further secured in independent experiments using established standard endpoint anti S IgG ELISAs (Fig. S6). Sera from immunized DO-HSC hosts, showed even better pseudovirus neutralization activity than those from immunized DY-HSC hosts (Fig. 3d). This confirmed that both young and old HSCs reconstitute very similar functional B cell systems in young RAG1^−/−^ hosts. We thus conclude that old HSCs retain competence, at least in a young environment, to reconstitute a youthful and functional B cell system that is associated with an efficient antigen-specific class-switch and IgG production after vaccination.

## Discussion

An impaired humoral immune response at the extreme old age is closely associated with an increased severity of pathogenic infections and decreased responsiveness to new antigens/pathogens. In particular, the recent COVID-19 pandemic as well as knowledge on seasonal Flu infections have shown that even very old people are able to elicit a broad spectrum of T and B cell responses upon vaccination that protect the majority from severe etiopathologies, though the efficacy to induce or maintain long-lasting antibody responses is indeed reduced [1, 16, 51]. However, such a direct relation between impaired immune responses and ageing-associated changes in the immune system is difficult to establish, as the immune responses are further critically influenced by broad range of pre-existing comorbidities [52, 53], chronical infections [54], but also by a multitude of individual changes and/or defects that could directly inhibit humoral immune responses independent of aging-associated remodeling of the immune system [55]. Interestingly, some defects in the old B-lineage such as the suppression of B cell lymphopoiesis by peripheral B cells remained reversible [56]. Depletion of B cells in old mice rejuvenated the newly reconstituting B cell system and tended to improve its immune competence [57, 58]. In contrast, B cells from aged individuals per se were not intrinsically defective to respond to stimulation and become antibody-secreting cells or to exert affinity maturation in response to immunization, suggesting that also B cell-extrinsic factors play a crucial role in the age-associated impairment in the humoral immunity [59, 60]. Little is known if and how ageing-associated changes, such as in old HSCs, in the old BM niche and/or in old secondary lymphoid structures [29, 30, 61–63] affect the production and functionality of B cells upon infection or vaccination. We here used a transplantation model to *de novo* reconstitute HSC-driven young and old B cell systems under the same conditions of a young environment to test immune competence by vaccination. The new key observations are: (i) in the same young environment, the overall reconstitution of CD45.1^+^ cells and CD19^+^ B cell subsets from DO-HSC compared to DY-HSC was reduced and thus directly related to old HSCs. (ii) both, DY-HSC and DO-HSC reconstituted very similar and largely unchallenged B cell systems in transplanted young RAG1^−/−^ hosts. (iii) old DO-HSC retain competence to reconstitute a youthful and functional B cell system in transplanted young hosts and vaccination with a recombinant SARS-CoV-2 S vaccine (NVX-CoV2373) induced very similar IgG^+^ S-antibody titers in both, DO-HSC and DY-HSC hosts associated with an efficient pseudovirus neutralization activity.

We here confirmed that the overall reconstitution of CD45.1^+^ cells and CD19^+^ B cells was reduced in DO-HSC as compared to DY-HSC hosts, although all HSCs must interact with the same young environment and target the bone marrow [28]. The frequencies of CD45.1^+^ donor derived long-term HSC (LT-HSC) fractions were very similar in the BM of DO-HSC and DY-HSC recipients. Both, DY-HSCs and DO-HSCs thus established in the young recipient BM with a similar efficacy, suggesting that the concomitant reduction in the *de novo* reconstitution of CD19^+^ B cells and CD45.1^+^ cells in DO-HSC vs. DY-HSC transplanted animals is directly related to changes in the differentiation program of old HSCs. CD19^+^ B cells developing from DY-HSC or DO-HSC differ in their gene expression signature [28]. These changes therefore likely affect primarily their propagation efficacy, but not their ability to reconstitute different B cell types and their response to a protein-based vaccine. Our findings further show that old HSCs, very similar to young HSCs, retain competence to reconstitute a youth-like and un-challenged B-cell system. Compared to old mice, this unchallenged DO-HSC-derived B-cell system largely lacked antigen-experienced B-cells such as ABC or memory B-cells.

Particularly in old mice, antibody responses critically depend on many factors, e.g., the antigen, the antigen delivery (e.g., DNA- or protein-based) and/or the immune modulatory adjuvant formulation used. The aged B cell system in old mice was able to distinguish between trimeric and monomeric antigen conformations: it respond better to trimeric than to monomeric antigen and a simple change in the vaccine delivery regimens like heterologous DNA-prime/protein-boost (DxP), but not protein-prime/DNA-boost (PxD) vaccination, was sufficient to unleash its reactivity to monomeric antigen [27]. This also confirmed that the old B cell system retain competence, at least in part, to respond to new antigens and vaccines, and that novel vaccination strategies might be sufficient to bypass functional limitations of an aged immune system and to efficiently induce high and protective antibody responses in old mice and humans [22–27]. We here used Nuvaxovid vaccine, a recombinant SARS-CoV-2 S-antigen delivered with Matrix-M adjuvants, because this recombinant vaccine induced antibody responses in old mice, meaning that the old B cell compartment is efficiently activated, even in the presence of multiple ageing-associated factors and/or cell types that might disturb the induction or maintenance of humoral immune responses.

In our studies, we detected higher numbers of germinal center (GC) B cells, the source of the high-affinity and class-switched antibodies [64], in DY-HSC and DO-HSC hosts as compared to ageing mice. This suggested that they might be spontaneously activated to some extend in the HSC-recipients by unknown mechanisms. However, considering the same young environment in transplanted RAG1^−/−^ hosts, we expect that the activation is very similar and not affected by the age of the transplanted HSCs. It is not known if and how variations in the number of GC B cells specifically affect priming of vaccine-induced antibodies, though the magnitude and quality of the germinal center (GC) response declines with age, among others due to a spatial dysregulation of T follicular helper cells [65]. We determined slightly higher GC B cell numbers in DY-HSC transplanted mice as compared with young mice, but both groups elicited very similar anti S IgG antibody titers. Higher GC B cell numbers, at least in this range, thus are not related to a higher vaccine induced anti S IgG antibody titers. The most intriguing finding of our study was that very similar anti S IgG titers in DO-HSC vs. DY-HSC transplanted mice showed a significant higher pseudovirus neutralization activity in the DO-HSC recipients. It is thus a possibility that anti S IgG antibodies primed in DO-HSC hosts show a better antigen affinity. However, we would expect that antibody affinity maturation is more efficient in DY-HSC hosts, because activation and affinity maturation of GC B cells by CD4^+^ helper T cells is more efficient in young mice and thus also in DY-HSC-transplanted hosts [65, 66]. Furthermore, we yet could not clarify if and how different antibody isotypes and/or IgG subclasses are differentially primed in vaccinated DO-HSC vs. DY-HSC recipients and affect pseudovirus neutralization.

In conclusion, our studies show that young and old HSCs reconstitute very similar unchallenged B cell systems that mediate antigen-specific IgG antibody responses by protein-based vaccination. This suggests that it is primarily age-related factors, and not HSCs per se, that influence the composition and functionality of the old B cell system.

## Electronic supplementary material

Below is the link to the electronic supplementary material.

Supplementary Material 1

## Data Availability

All data relevant to this study are included in the article or uploaded as supplemental files. The raw data supporting the conclusions of this article will be made available by the authors upon reasonable request.

## Abbreviations

ABC: Age-associated B cell
APCL: Allophycocyanin
ASC: Antibody secreting cells
BM: Bone marrow
DMEM: Dulbecco´s modified eagle medium
DO: Donor old
DY: Donor young
FCM: Flow cytometry
FCS: Fetal calf serum
FOB: Follicular B cell
GC B: Germinal center B cell
HRP: Horseradish peroxidase
HSC: Hematopoietic stem cells
IgG: Immunglobulin G
LT-HSC: Long-term hematopoietic stem cells
MZB: Marginal zone B cell
OPD: o-Phenylenediamine Dihydrochloride
PBS: Phosphate buffered saline
PE: Phycoerythrin
PVNT50: 50% pseudovirus neutralizing titer
QC: Quality control
RBD: Receptor binding domain
RLU/s: Relative light units per second
S-antigen: SARS-CoV-2 surface antigen
SMB: Switched memory B cell
SPF: Specific pathogen free
ST-HSC: Short-term hematopoietic stem cells
VSV: Vesicular stomatitis virus
WBC: White blood cell
