# Supplementary material for: Old hematopoietic stem cells retain competence to reconstitute a youthful B cell system that is highly responsive to protein-based vaccination
Source: Immun Ageing. 2025 Apr 5;22:14. doi: 10.1186/s12979-025-00507-x (PMC11971919; doi:10.1186/s12979-025-00507-x)
Supplement: Supplementary file 1 — Supplementary Material 1 [file 12979_2025_507_MOESM1_ESM.docx]

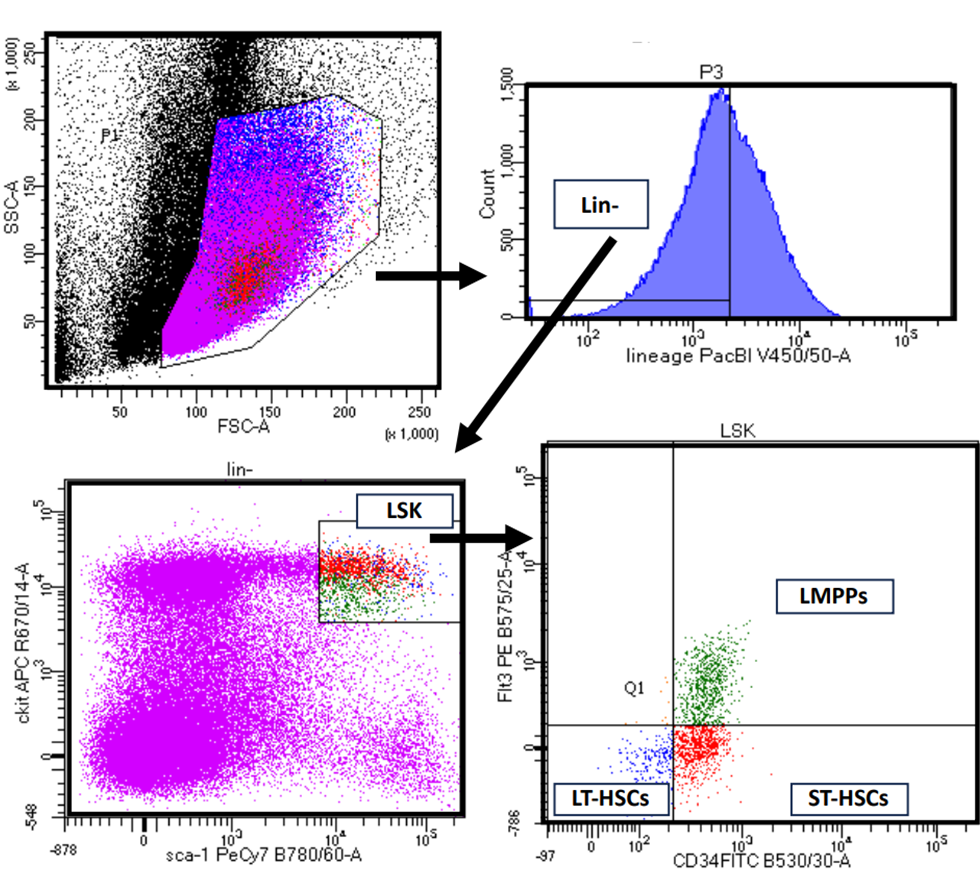

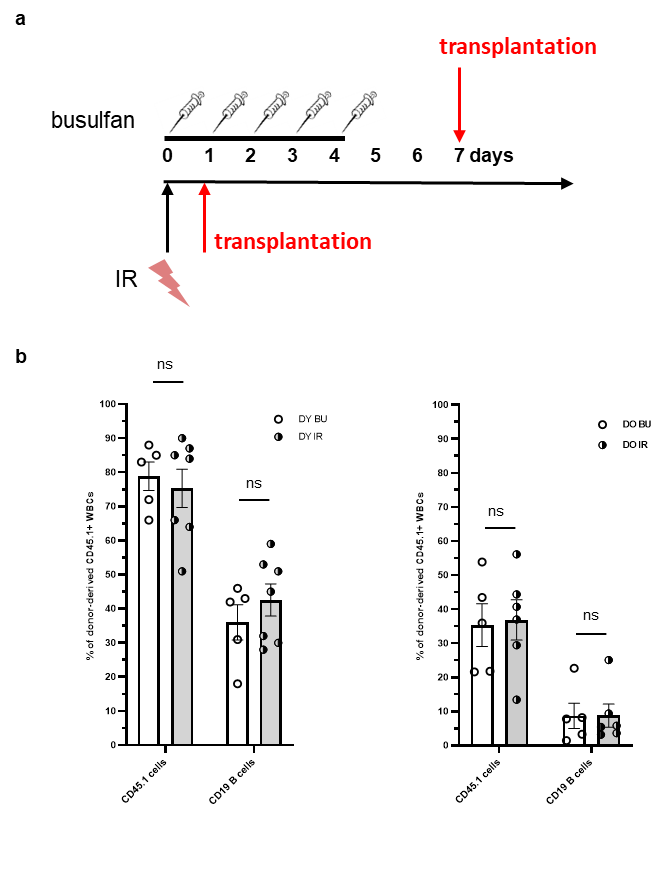


**Supplementary Figure S1.**

**Gating strategy for donor-derived LT-HSCs for transplantation into RAG1^-/-^ recipient mice.**

After antibody-mediated depletion, lineage markers were used to further exclude differentiated cells. Lineage negative cells (Lin^-^) were further selected for expression of c-Kit and Sca-1 as selection markers for progenitor cells (LSK, Lin^–^ Sca-1^+^ c-Kit^+^). These were further distinguished into long-term hematopoietic stem cells (LT-HSCs), short-term hematopoietic stem cells (ST-HSCs) and lympho-myeloid primed progenitors (LMPPs) via Flt3 and CD34 gating.

**Supplementary Figure S2.**

**HSC-transplantation into irradiated vs busulfan-treated RAG1^-/-^ hosts.**

**(a)** Schematic presentation of the experimental setup: RAG1^-/-^ mice were either irradiated (IR) or pretreated for five consecutive days with Busulfan (BU) followed by transplantation of CD45.1^+^ HSCs at day one or seven, respectively. HSCs used were isolated either from young (DY-HSCs; 3 months; n=5-7) or old (DO-HSCs; 23 months; n=5-6) immune-competent mice**.** The percentage of CD45.1^+^ donor cell and CD19^+^ B cell contribution to total white blood cells (WBC) was determined in the peripheral blood of DY-HSC (DY-HSC/IR vs DY-HSC/BU; left panel) or DO-HSC hosts (DO-HSC/IR vs DO-HSC/BU; right panel) at 18 weeks post transplantation. Statistical significance between the indicated groups was determined using the unpaired students t-test. ns, not significant. Mean values + SD are shown.


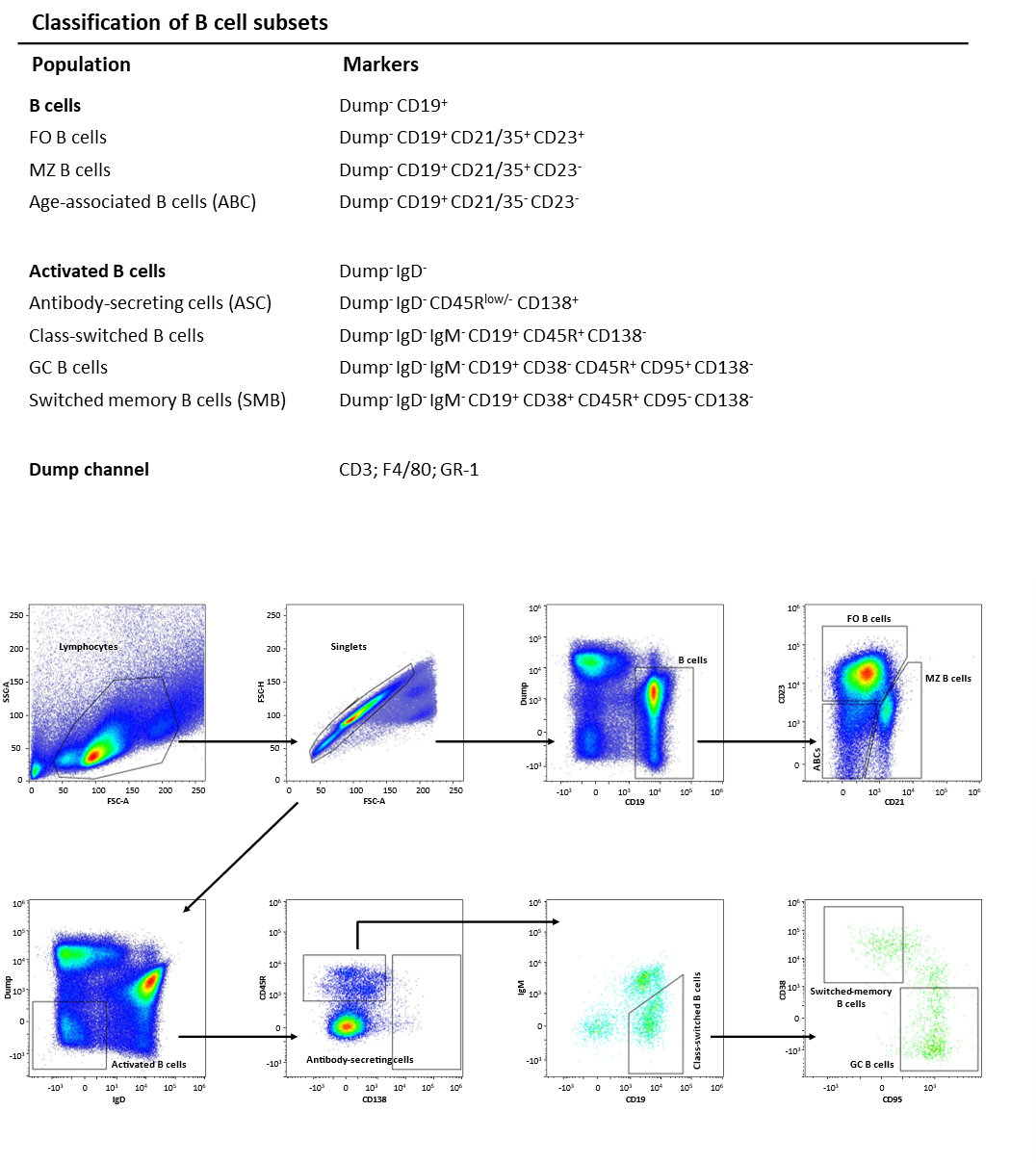


**Supplementary Figure S3.**

**Classification and gating of B cell subsets.**

B cell subsets with well-established surface marker profiles were analyzed by FCM.


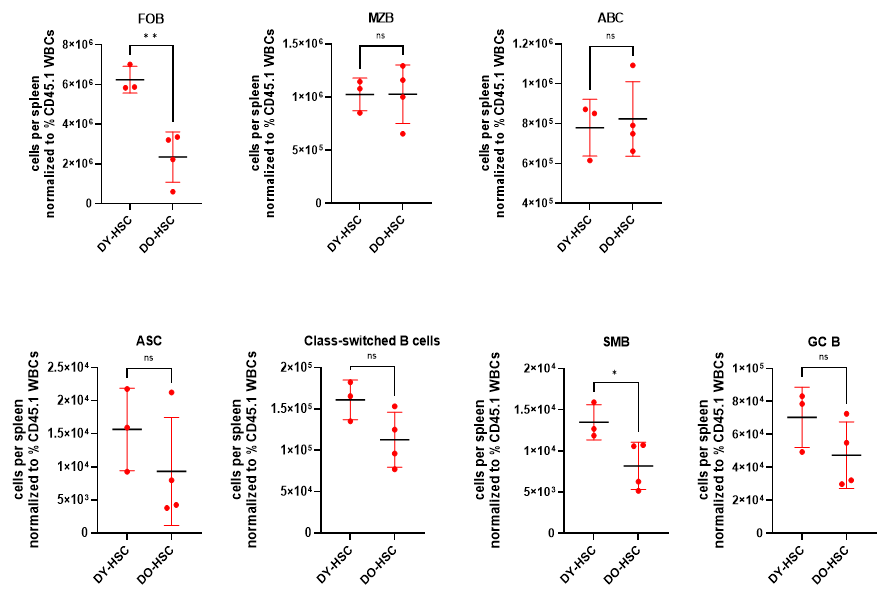


**Supplementary Figure S4.**

**Determination of B cell subsets normalized to the age-dependent HSC-driven reconstitution efficacy.**

The number of B cell subsets in DY-HSC and DO-HSC transplanted hosts was normalized to the overall HSC-driven reconstitution efficacy of CD45.1^+^ cells at 18 weeks post transplantation (see Fig. 1c), i.e., an average of 89% vs 58% for DY-HSC and DO-HSC hosts, respectively. The B cell numbers were calculated for an engraftment efficacy of 100% CD45.1^+^ cells. Statistical significance between the indicated groups was determined using the unpaired students t-test. P-values smaller than 0.05 were considered as statistically significant and indicated with asterisks in the graphs (p<0.05*, p<0.01**). ns, not significant. Mean values + SD are shown.


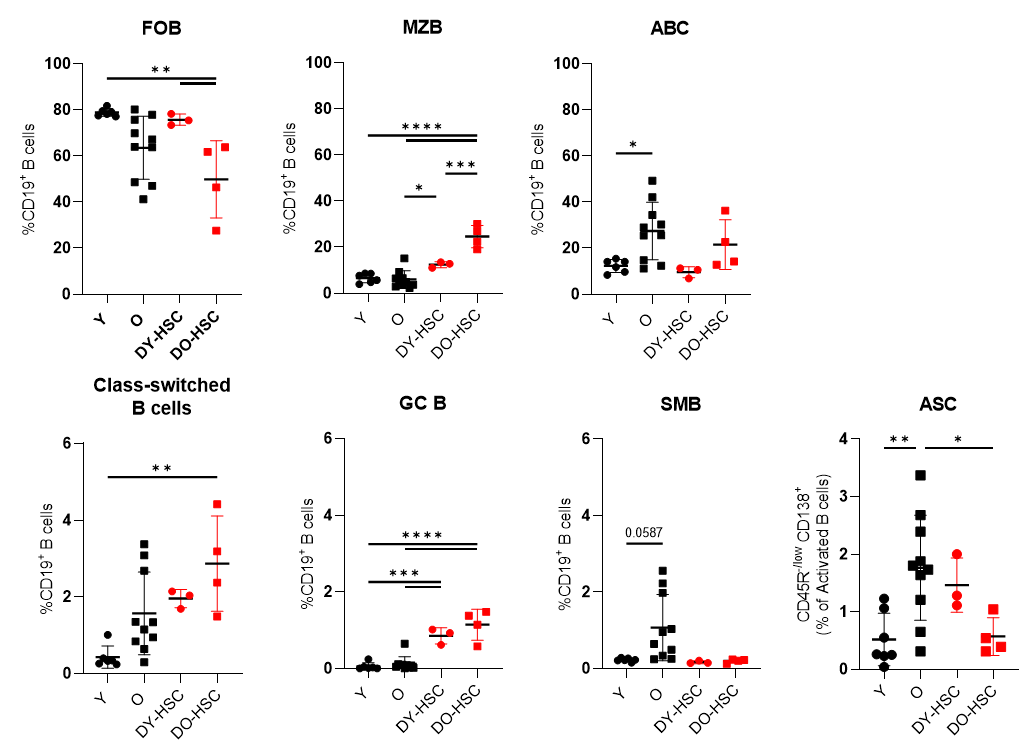
**Supplementary Figure S5.**

**Frequencies of B cell subsets shown in Fig. 2.**

The numbers of the different B cell subsets in the spleen of young (Y) and old (O) mice, and DY-HSC and DO-HSC transplanted mice (shown in Fig.2c-f) were used to calculate their relative frequencies within the CD19^+^ B cell pool (Fig.2a,b) or, depending on the marker profile of ASCs (see Fig.S3), within the activated B cell pool. Statistical significance between the indicated B cell populations was determined using one-way ANOVA with Tukey,s multiple comparison test. p<0.05*, p<0.01**, p<0.001***. p<0.0001****. If not indicated, the differences do not reach statistical significance. Mean values + SD are shown.

**Supplementary Figure S6.**

**Determination of S-specific IgG^+^ antibody titers by endpoint ELISA.**

DY-HSC and DO-HSC-transplanted mice were immunized at 18 weeks post transplantation (n=3) (at day 0 and 22) with 1 µg recombinant Novaxovid. S-specific IgG antibody titers were determined at day 14 after the final immunization by endpoint ELISA as described in the M&M section. Statistical significance between the groups was determined using the Mann-Whitney test. ns, not significant. Mean values + SD are shown.
